# Expression of precipitating factors of pruritus found in humans in an imiquimod-induced psoriasis mouse model

**DOI:** 10.1016/j.heliyon.2019.e01981

**Published:** 2019-06-20

**Authors:** Nobuo Oishi, Hiroki Iwata, Naotomo Kambe, Noriko Kobayashi, Kazuko Fujimoto, Hiromi Sato, Akihiro Hisaka, Koichi Ueno, Katsunori Yamaura

**Affiliations:** a Division of Social Pharmacy, Center for Social Pharmacy and Pharmaceutical Care Sciences, Faculty of Pharmacy, Keio University, 1-5-30 Shibakoen, Minato-ku, Tokyo, 105-8512 Japan; b Department of Dermatology, Kansai Medical University, 2-5-1 Shin-machi, Hirakata, Osaka 573-1010, Japan; c Laboratory of Clinical Pharmacology and Pharmacometrics, Graduate School of Pharmaceutical Sciences, Chiba University, 1-8-1 Inohana, Chuo-ku, Chiba, 260-8675 Japan; d Center for Preventive Medical Science, Chiba University, 1-8-1 Inohana, Chuo-ku, Chiba, 260-8675, Japan

**Keywords:** Neuroscience, Immunology, Pruritus, Mast cell, Neurotrophin, Psoriasis, Neuropeptide

## Abstract

**Aims:**

To use a mouse model of imiquimod-induced psoriasis to investigate the relationship between pruritus and mast cells, nerve growth factor (NGF) and endogenous pruritogenic peptides, which are highly expressed in the skin of psoriasis patients.

**Main methods:**

We developed a mouse model of imiquimod-induced psoriasis and measured the frequency and duration of the model animals’ self-scratching behavior using the SCLABA^®^-Real real-time scratch counting system. We then harvested the ears and subjected them to toluidine blue staining and real-time PCR.

**Key findings:**

Topical application of imiquimod increased the Psoriasis Area and Severity Index score as well as the frequency and duration of self-scratching. Regarding internal factors, increases in mast cells number and mRNA expression of NGF and endogenous pruritogenic peptide precursor were confirmed.

**Significance:**

Self-scratching behavior is accompanied by increased number of mast cells and expression of NGF and endogenous pruritogenic peptides in our imiquimod-induced psoriasis model. The expression of these factors was consistent with the features in patients with pruritic psoriasis, suggesting that our model reflects at least some of the precipitating factors of pruritus found in humans.

## 1 Introduction

Pruritus is defined as “an unpleasant sensation that causes a desire to scratch”, and occurs on the skin, mucous membranes, and cornea. Itching is considered to be a biological defense sensation that was originally intended to remove parasites and other foreign bodies from the body surface by inducing a scratching motion [1]. Itching also causes an aggravation of the causative skin condition as well as sleep disorders and diminished concentration and judgement. Therefore, treatments that takes the alleviation of itching into consideration are requisite.

According to a 2009 report by Prignano et al., pruritus occurs in about 85% of psoriasis patients [2]. An important finding linking psoriasis and pruritus is the Koebner phenomenon, in which external stimuli results in psoriatic lesions [3]. Uncontrolled itching and scratching increase the risk of enlarging existing psoriatic lesions; patient education thus encourages psoriasis patients to avoid stimuli to their skin, including scratching.

The expression of pruritogenic mediators in psoriatic lesions has been investigated [4, 5, 6]. Mast cells store granules containing histamine, serotonin, substance P, and tryptase. These mediators are released with degranulation, and induce pruritus via their receptors on the C-fiber. Nerve growth factor (NGF) lowers the itch threshold via neuronal elongation, and thus contributes indirectly to the aggravation of pruritus.

Enkephalin is produced by keratinocytes or fibroblasts and its fragments include neuropeptides such as opioid peptides and endogenous pruritogenic peptide bovine adrenal medulla 8–22 (BAM8-22). BAM8-22 acts as a ligand of MrgprC11 and transmits itch in a histamine- and opioid-independent manner. These factors are associated with pruritus in psoriasis patients and could be used as a pruritus marker.

Quantitative evaluation of pruritus is considered difficult because it is a subjective symptom. Currently, the most commonly used clinical method is the visual analogue scale (VAS), which was originally a system for pain evaluation and its application has now been expanded to include pruritus [7]. However, the score is relative; each individual would have a different standard and the degree of variation would depend on the population. It was thus necessary to develop a more objective method for evaluation of pruritus, and in 1995,

Kuraishi et al. established a method that focused on the self-scratching behavior of mice using their hind paws [8]. An established animal model of psoriasis was developed using imiquimod (IMQ), a TLR7 agonist [9]. Wong et al. confirmed pruritus in IMQ-induced psoriasis mouse model and found that vascular endothelial growth factor expression and neuronal elongation are involved in the mechanism underlying induction of pruritus [10]. Meanwhile, Satake et al. showed that neuronal elongation by NGF is involved in pruritus in IMQ-induced psoriasis mouse model, and that the opioid antagonist naltrexone and the histamine H1 receptor antagonist olopatadine contribute to the suppression of pruritus [11].

Based on these findings, we determined the expression levels of factors that are enhanced or promoted in psoriasis patients with pruritus *i.e*. mast cells, tryptase, NGF, and endogenous pruritogenic peptides in IMQ-induced psoriasis mice, in order to investigate how closely this animal model mimics human pruritic psoriasis.

## 2 Methods

### 2.1 Animals

Female BALB/c mice (8 weeks old; Japan SLC) were kept in an environment with constant temperature and humidity (24 ± 1 °C and 60 ± 5%, respectively) with a 12-h light-dark cycle. The animals were given MF pellets (Oriental Yeast Co., Ltd., Tokyo, Japan) and tap water *ad libitum* and allowed to acclimatize for about 1 week before the experiment. This study was approved by Chiba University Animal Care Committee and Keio University Institutional Animal Care and Use Committee.

### 2.2 Reagents

Propeto^®^ (white petrolatum) was purchased from Maruishi Pharmaceutical Co., Ltd., Osaka, Japan and Beselna cream 5% (5% w/w IMQ) was provided by Mochida Pharmaceutical Co., Ltd., Tokyo, Japan.

### 2.3 Developing the IMQ-induced psoriasis-like dermatitis model and pruritus evaluation

A mouse model of psoriasis was generated as described by van der Fits *et al.* [9] Briefly, 7.5 μL/ear Propeto or Beselna cream were applied topically once a day to the inner ear of the animals from day 0–5. Measurement of ear swelling and Psoriasis Area and Severity Index (PASI) score for mice were taken on days 0, 2, 4, and 6 under blinded conditions. A dial thickness gauge (Mitutoyo M3 Center, Aurora, IL) was used for measurements of ear thickness. The average thickness of the right and left ears were compared to that of day 0 for the calculation of ear thickening. On day 5, following a 1-h acclimatization period, the frequency of scratching behavior was recorded using the SCLABA^®^-Real (Noveltec Inc., Kobe, Japan) real-time scratch counting system from 9 to 21 h after topical application of the creams. Locomotor activity was also measured from image data. During acclimatization, each chamber was supplied with 1/6 cup of DietGel^®^ 76A (water-containing feed; SLG). The conditions of analyses were set as θc = 27, τ0 = 30, τ1 = 70 and τ2 = 150. The animals were sacrificed after dermatitis severity assessment on day 6 and the ears were harvested. All right ears were frozen in liquid nitrogen and kept at -80 °C to be used for real-time PCR. All left ears were embedded in optimal cutting temperature (OCT) compound and frozen in liquid nitrogen to be used for toluidine blue (TB) staining.

### 2.4 Toluidine blue staining

The left ears were embedded in OCT compound (Sakura Finetek, Tokyo, Japan), frozen and then sectioned to 10 μm with the Leica CM3050S cryostat (Leica, Wetzlar, Germany). Sections were mounted on glass slides (Matsunami Glass Ind., Ltd., Osaka, Japan), dried overnight, washed in water to remove the OCT compound, and then stained with 0.05% Toluidine Blue Solution (pH 4.1; Wako Pure Chemical Industries, Osaka, Japan) for 10 min. After staining, sections were washed briefly in water, subjected to differentiation and dehydration 3 times with 95% ethanol, and another 3 times with 99% ethanol, cleared in xylene (Wako) 3 times, and then sealed in Entellan^®^ new cover slipper for microscopy (Merck KGaA, Darmstadt, Germany). After air-drying, images of the sections were acquired in the same orientation under a microscope and mast cells were counted under blinded conditions. Six fields of view were randomly selected per sample, and the average of these fields was taken as the number of cells.

### 2.5 RNA extraction and real-time PCR

The right ears were homogenized in RNAzol^®^ RT (Molecular Research Center, Inc., Cincinnati, OH), mixed with water, left to stand, and then centrifuged in order to precipitate out the DNA and protein. RNA was then precipitated by mixing 2-propanol (Wako), leaving the sample to stand, and then centrifuging. The RNA pellet was washed in 75% ethanol (Wako), centrifuged and then dissolved in RNase-free H_2_O. ReverTra Ace^®^ qPCR Master Mix (Toyobo Co., Ltd., Osaka, Japan) and primers of to-be measured factors (Eurofins Genomics Toronto, ON) were used to reverse-transcribe cDNAs from the mRNAs in a PCR Thermal Cycler (Takara Bio Shiga, Japan). Each RNA solution was diluted to 1000 ng/10 μL. The thermal cycle program was 15 min at 37 °C, 5 min at 50 °C, and 5 min at 98 °C, and cooling to 4 °C.

The PCR reaction was carried out using SYBR^®^ Premix Ex Taq (Perfect Real Time; Takara Bio) and Step One^TM^ Real Time PCR System (Applied Biosystems, Foster, CA). Total reaction volume was adjusted to 20 μL following the manufacturer's protocol and the thermal cycle program was 10 s at 95 °C, 50 cycles of 5 s at 95 °C, and 31 s at 60 °C, followed by a gradual increase from 60 °C to 95 °C in 0.5 °C increments to melt the PCR product. Expression levels of RNA are presented relative to the internal control GAPDH.

### 2.6 Statistical analyses

All data are shown as the average ±standard error. Analyses were performed using Stat Light 1997 (Yukms) software. We performed two samples parametric tests, which were Aspin Welch t-test, Student's t-test or Mann Whitney U-test; statistical significance was set to p < 0.05.

## 3 Results

### 3.1 PASI scores, scratching frequency and locomotor activity

**Fig. 1:**
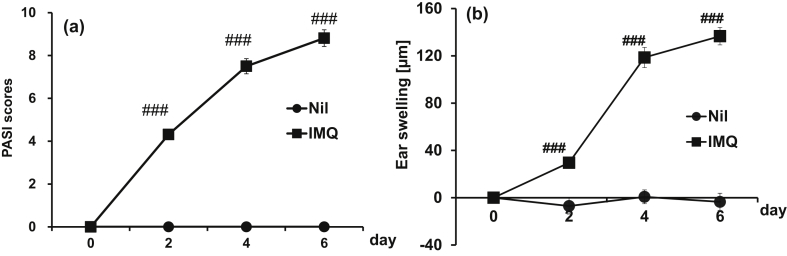
Changes in total PASI scores in IMQ-treated mice. Either white petrolatum or 5% IMQ cream was applied to the ears of mice repeatedly for 6 consecutive days. (a) PASI score and (b) ear swelling evaluation was done 24 h after each IMQ challenge on day 0, 2, 4, and 6. Scaling, erythema, and thickening of both ears was scored on a scale from 0 to 4 and total PASI score (Scaling plus erythema plus thickening) was determined. Data are expressed as the mean ± S.E.M. for n = 4 mice. ###p < 0.001 compared to Null mice ((a) Mann Whitney U-test, (b) Student's t-test or Aspin Welch t-test).

**Fig. 2:**
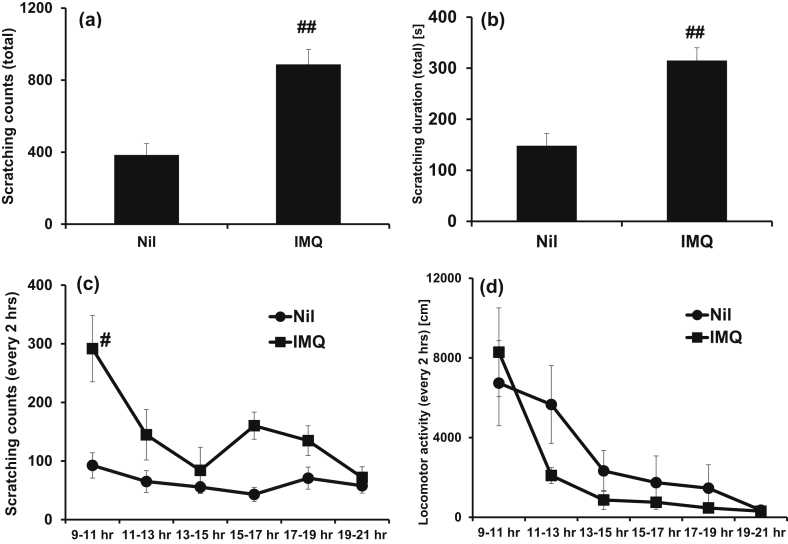
Scratching behavior and locomotor activity in IMQ-treated mice. Either white petrolatum or 5% IMQ cream was applied to the ears of mice repeatedly for 6 consecutive days. On day 5–6, (a) and (c) scratching counts, (b) scratching duration, and (d) locomotor activity of mice were measured by SCLABA^®^-Real system after topical application of 5% IMQ cream for 12 h. Data are expressed as the mean ± S.E.M. for n = 4 mice. ##p < 0.01 compared to Nil mice (Student's t-test or Aspin Welch t-test).

Over time, the PASI score and ear swelling increased in the IMQ group; compared to the Nil group that did not receive IMQ treatment, their scores were higher on days 2, 4, and 6 (Fig. 1a and b). Frequency and duration of the self-scratching behavior of mice were set as indices of pruritus. Locomotor activity was recorded in parallel to further confirm the effect of topical IMQ application on mice behavior. Mice are nocturnal animals, and so recording was done in the dark phase. Self-scratching frequency and duration in the IMQ group during the total recording time (12 h) was 2.2 and 2.1 times that of the Nil group, respectively (Fig. 2a and b).

Further observations over time showed that both the Nil and IMQ groups had their peak self-scratching frequency between 9 and 11 h after topical application. The peak frequency of the IMQ group was about 3.2 times that of the Nil group. This difference had mostly disappeared at 19 h after topical application (Fig. 2c). Peak locomotive activity was observed between 9 and 11 h after topical application in both groups; there was no difference between the groups at any timepoints (Fig. 2d).

### 3.2 TB staining

**Fig. 3:**
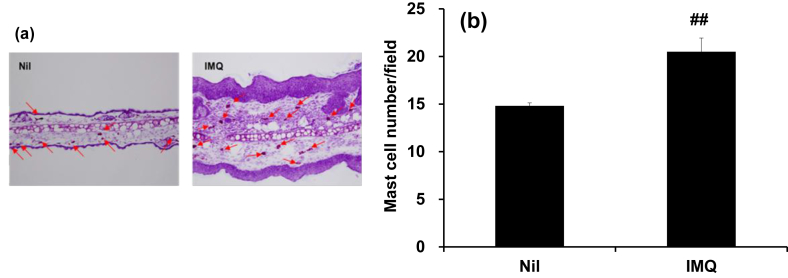
Mast cell number in ear tissue of IMQ-treated mice. After evaluation of PASI score on day 6, mice were sacrificed and their left ears were sampled. Ear samples were embedded in O.C.T. compound, frozen in liquid nitrogen, and sliced at 10 μm and stained with TB for from Nil and IMQ mice. (a) Representative skin samples on microscopy (arrows represent mast cell-positive sites). (b) Mast cell number per field of microscopic assessment of mast cells. Data are expressed as the mean ± S.E.M. for n = 4 mice. ##p < 0.01 compared to Nil mice (Aspin Welch t-test).

Mast cells release pruritogens, and thus contribute to pruritus in atopic dermatitis and contact dermatitis. In order to confirm the changes in the number of mast cells in the tissue, mouse ear tissues were subjected to TB staining. Mast cells per field of view increased to around 1.4 times in the IMQ group compared to the Nil group (Fig. 3a and b).

### 3.3 Ear tissue mRNA expression

**Fig. 4:**
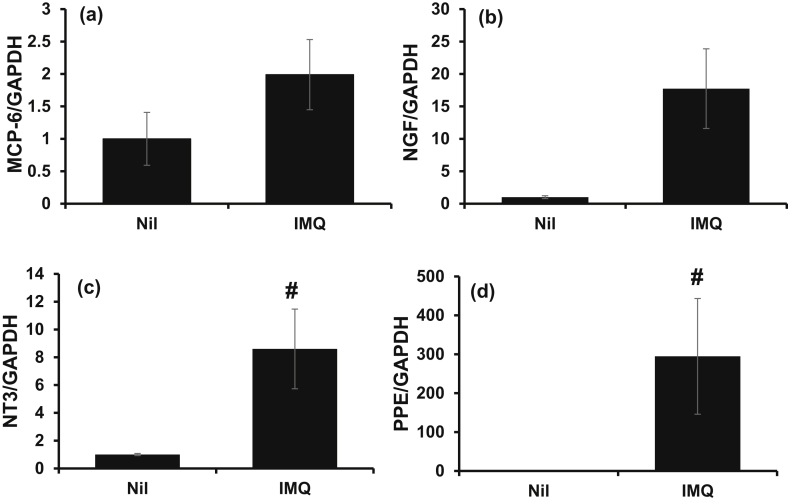
mRNA expression in ear tissue of IMQ-treated mice. After evaluation of PASI score on day 6, mice were sacrificed and their right ears were sampled. Total RNA was extracted from each ear, and expression of (a) MCP-6, (b) NGF, (c) NT3 and (d) PPE was determined by quantitative Real time-PCR. Data are expressed as the mean ± S.E.M. for n = 4 mice. #p < 0.05 compared to Nil mice (Mann Whitney U-test).

mRNA expression of mast cell protease-6 (MCP-6), a mast cell-derived tryptase, increased about 2 times in the IMQ group compared to the Nil group, although this was not statistically significant. This supported the increase in mast cell number observed on TB staining (Fig. 4a). NGF expression was about 18 times higher in the IMQ group than the Nil group (Fig. 4b), which is consistent with previous findings [10, 11]. Similarly, expression of another neurotrophic factor neurotrophin3 (NT3) was also 8.6 times higher in the IMQ group compared with the Nil group (Fig. 4c); this differed from the report by Nakamura et al. in humans [4]. For enkephalin precursor preproenkephalin (PPE), which is highly expressed in the skin of psoriasis patients, expression in the IMQ group was about 295 times higher than the Nil group (Fig. 4d); this was consistent with the report by Slominski et al. in humans [6].

### 3.4 Scatter plots for each mouse

**Fig. 5:**
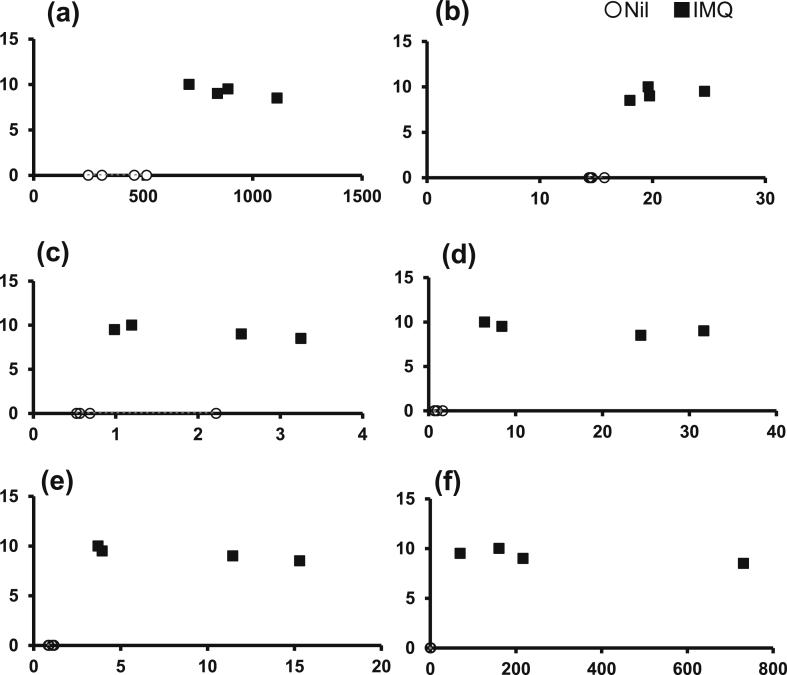
Scatter plots for each mouse (PASI score and other parameters). The correlation coefficients (r) between PASI scores and (a) scratching count, (b) mast cell count, and the expression levels of (c) MCP-6, (d) NGF, (e) NT3, and (f) PPE were 0.86, 0.86, 0.46, 0.57, 0.68 and 0.57, respectively (Y-axis, PASI score; X-axis, each factor).

**Fig. 6:**
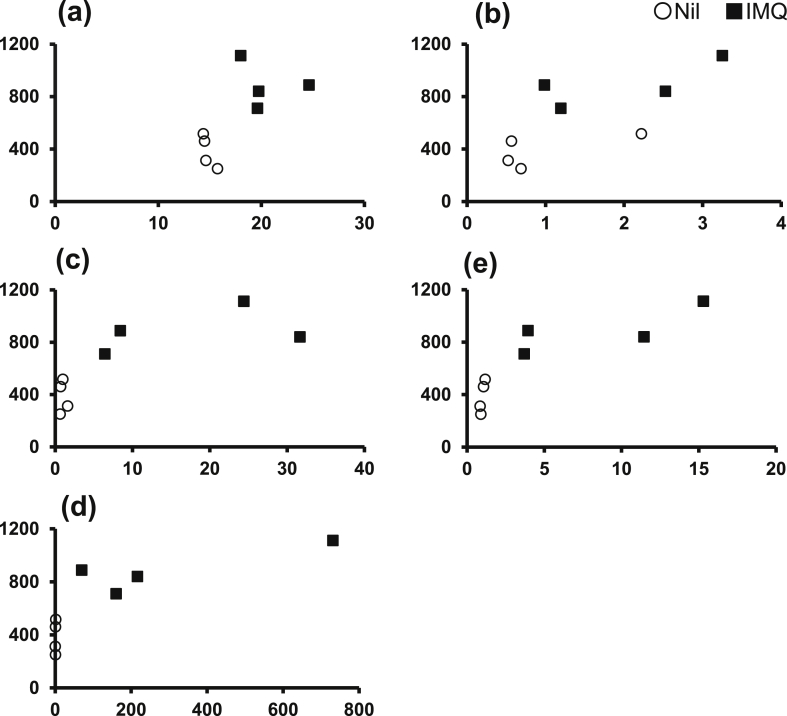
Scatter plots for each mouse (scratching count and other parameters). The correlation coefficients (r) between scratching count and (a) mast cell number, and the expression levels of (b) MCP-6, (c) NGF, (d) NT3 and (e) PPE were 0.68, 0.73, 0.80, 0.85 and 0.80, respectively (Y-axis, scratching count; X-axis, each factor).

Scatter plots for each mouse are shown in Figs. 5 and 6. The correlation coefficients (r) between PASI scores and scratching count, mast cell count, and the expression levels of MCP-6, NGF, NT3 and PPE were 0.86, 0.86, 0.46, 0.57, 0.68 and 0.57 respectively (Fig. 5). Similarly, the correlation coefficients (r) between scratching count and mast cell number, and the expression levels of MCP-6, NGF, NT3 and PPE were 0.68, 0.73, 0.80, 0.85 and 0.80, respectively (Fig. 6).

## 4 Discussion

Topical application of IMQ increased PASI scores and self-scratching behavior in mice (Figs.1a, 1b, 2a and 2b). This was consistent with previous studies [10, 11], and thus confirmed that our animal model was representative human psoriasis, including pruritus. Moreover, our study showed for the first time that the increased mast cell number and enhanced neuropeptide expression observed in psoriasis patients are also reproduced in this mouse model.

Mast cell number increased by about 40% in the IMQ group (Fig. 3a and b), which is consistent with the increase seen in patients with pruritic psoriasis [4]. MCP-6 is a mouse mast cell-derived tryptase. Although the detailed mechanism is unknown, this tryptase is involved in inflammation in the mouse arthritis model [12] and is considered an index of mast cell degranulation. In our study, MCP-6 mRNA showed a tendency to increase, supporting the increase in mast cell number (Fig. 4a). In the NC/Nga mouse model of atopic dermatitis, disease onset increased mast cell number 5-fold [13]. Taken together, the contribution mast cells to pruritus in psoriasis may not be as predominant as in atopic dermatitis.

The source of NGF in the skin is said to be keratinocytes and nerve cells [14]. NGF was found to degranulate mast cells directly *in vitro/in vivo* [15, 16], suggesting its suspected involvement in pruritus. In our investigation, NGF expression was enhanced (Fig. 4b), which concurs with the observation in psoriasis patients [4]. NT3 is a neurotrophin that is unrelated to NGF, and its detailed mechanism of action is largely unknown. The relationship of NT3 to mast cells has been suggested, as it is involved in the survival and function of mast cells in mouse embryos [17] and coexists with NGF and BDNF in mast cells and is released with degranulation [18]. NT3 production was promoted in the IMQ-induced psoriasis mouse model in our study (Fig. 4c); however, this result differed from the features seen in psoriasis patients [4]. This suggests an interspecies difference between mice and humans in the expression patterns of neurotrophic factors and degranulation substances from the mast cells.

The mRNA expression of enkephalin precursor PPE was promoted by topical application of IMQ (Fig. 4d), which supports the findings of Slominski *et al.* reported that naltrexone, an opioid antagonist, and olopatadine, a histamine H1 receptor antagonist, have suppressed pruritus in their mouse model of psoriasis [6], indicating that histamine and opioid pathways are involved in pruritus induction in psoriasis. Meanwhile, BAM8-22, a neuropeptide derivative of PPE, transmits itch in a histamine- and opioid-independent manner. Our findings thus indicate that pruritus in psoriasis is the result of the complex intertwining of multiple pathways and factors. It is plausible that the contribution of BAM8-22 to this pathology could be clarified if itching could be suppressed by inhibiting the involved pathways. However, such inhibitors are yet to be developed, and presently, there is only one report on a candidate compound, the MrgprC11 antagonist [19].

As limitations of this study, the factors we measured were limited and the contribution of various factors, including not only what we measured but also other pruritogenic substances to IMQ-induced pruritus was not shown strictly. Accordingly, further studies are needed to clarify the IMQ-induced psoriasis mouse model reflect more various factors of pruritus found in humans.

## 5 Conclusion

We have reported that self-scratching behavior in IMQ-induced psoriasis model mice is accompanied by increased number of mast cells and expression of NGF and neuropeptides, which is approximately representative of human psoriasis patients. This animal model could be useful in elucidating the mechanism of psoriatic pruritus and in evaluating anti-pruritus drug efficacy.

## Declarations

### Author contribution statement

Nobuo Oishi: Conceived and designed the experiments; Performed the experiments; Analyzed and interpreted the data; Wrote the paper.

Hiroki Iwata, Naotomo Kambe: Performed the experiments.

Noriko Kobayashi, Kazuko Fujimoto, Hiromi Sato, Akihiro Hisaka: Analyzed and interpreted the data.

Koichi Ueno: Contributed reagents, materials, analysis tools or data.

Katsunori Yamaura: Conceived and designed the experiments; Performed the experiments; Analyzed and interpreted the data; Contributed reagents, materials, analysis tools or data; Wrote the paper.

### Funding statement

This work was supported in part by Grants-in-Aid for Scientific Research from the Japan Society for the Promotion of Science (grant 15K08666).

### Competing interest statement

The authors declare no conflict of interest.

### Additional information

No additional information is available for this paper.
